# Electron Scattering in Conventional Cell Flask Experiments and Dose Distribution Dependency

**DOI:** 10.1038/s41598-019-57029-y

**Published:** 2020-01-16

**Authors:** Jeremy S. Haskins, Stephen K. Martinez, Madison Engstrom, Mami Murakami, Takashi Mori, Del Leary, Takamitsu A. Kato

**Affiliations:** 1 Department of Environmental & Radiological Health Sciences, Colorado State University, Fort Collins CO, 80523 USA; 2 United Department of Veterinary Medicine, Faculty of Applied Biological Sciences, Gifu University, 1-1 Yanagido, Gifu, 501-1193 Japan; 3 Center for Highly Advanced Integration of Nano and Life Sciences, Gifu University (G-CHAIN), 1-1 Yanagido, Gifu, 501-1193 Japan

**Keywords:** Radiotherapy, Radiotherapy

## Abstract

Electron beam therapy (EBT) is commonly used for treating superficial and subdermal tumors. Previous cellular radiosensitivity research using EBT may be underestimating the contribution from flask wall scattering and the corresponding dose distribution. Single cell suspensions of Chinese hamster ovary (CHO) cells were plated on flasks and irradiated with 3, 4, 7, 9, and 18 MeV energy electron beams from two different institutions, and the spatial locations of surviving colonies were recorded. Gafchromic film dosimetry and Monte Carlo simulations were carried out to determine the spatial electron scattering contribution from the flask walls. Low electron irradiation resulted in an uneven surviving colony distribution concentrated near the periphery of the flasks, while spatial colony formation was statistically uniform at energies above 7 MeV. Our data demonstrates that without proper dosimetric corrections, studies using low energy electrons can lead to misinterpretations of energy dependent cellular radiosensitivity in culture vessels, and radiotherapeutic applications.

## Introduction

Electron beam therapy (EBT) is an effective radiotherapy for treating superficial and subdermal tumors such as skin lesions, melanomas, lymphomas, and a variety of other skin-deep malignancies. Total skin electron beam irradiation techniques were conceived as early as the 1960s to 1980s^1^, with little change since. Currently, it is estimated that more than one million American’s are treated with EBT. Each year, 14 million new cases^2^ of cancer are diagnosed and the incidence of cancer is expected to increase steadily. Electron beam therapy is often combined with surgical resection to improve the local control^3,4^. The typical electron energy used for the electron beam therapy is between 2 and 25 MeV with various depth dose characteristics and scatter properties that are chosen depending on the depth and size of the tumor.

Although the physical dose distribution of electron beam therapy is well understood in water or homogeneous tissue equivalent material, biological studies using electron beams has limited supporting data. In order to investigate the comparative biological effects of electron irradiation, early studies were carried out via beta-emitting radioisotopes. These studies found electron beam radiation has qualities similar to low linear energy transfer (LET) photon radiation. Clinically relevant electron energies have approximately the same relative biological effectiveness as photons^5,6^.

Radiographic film is a prominent tool for evaluating planar dose distribution in both photon^7^ and electron^8,9^ modalities. Film has high spatial resolution and provides a permanent recording of the integrated dose distribution. It is widely used in dosimetric comparisons to calculated dose for external beam radiation quality assurance^10^. The optical density of film changes in a predictable way with dose and calibration curves over a range of doses can be made to characterize the response to novel dose distributions^11^.

Electron transport calculation also has a substantial role in the development of electron beam therapy^12^. Monte Carlo calculations are the most accurate method of modeling electron transport, and are rising to prominence with advancing computation power. Previously, the combination of film and Monte Carlo modeling were used to analyze scatter from medical devices^13^. Cell culture vessels have been analyzed previously using film and analytical electron transport techniques for clinical megavoltage and kilovoltage photon beams^14^. Large variations in absorbed dose caused by the irregular geometry of the vessels were observed prompting caution in radiobiological experiments.

This study originally aimed to investigate radiosensitivity of different energies of electrons with Chinese hamster ovary cells. Surprisingly, uneven surviving colony distribution on the flasks was observed at low energies of electrons. Therefore, we hypothesized, that electrons delivered at different energies have different scattering characteristics that interact with the flask wall and can deliver uneven dose distribution to the flask as a whole. In this paper, we investigated uneven survivors in the cell culture vessels, uneven dose distribution from DNA damage, corresponding film dosimetry, and the comparative scattering distributions from Monte Carlo simulations.

## Materials and Methods

### Irradiation

At Colorado State University (CSU, Fort Collins, CO), a MV linear accelerator (Varian Trilogy, Varian, Palo Alto, CA) used for radiotherapy on veterinary oncology patients routinely accelerated electron at 4, 9, and 18 MeV and irradiated cells in cell culture vessels from directly above. The field of irradiation for these experiments was 25 cm × 25 cm chosen to be considerably larger than the surface area of the flasks. This was done to minimize field edge effects over the irradiated cells and produce a uniform dose over the cell culture area within the flask. The dose for each energy was equal at 2 mm depth to account for the media thickness and knowledge that the cells were at the surface of flask below the media. This was done by using percent depth dose (PDD) data and adjusted to give an equal dose for each energy. The dose rate was approximately 10 Gy per min. Irradiation was carried out at room temperature. For each experiment the flasks were placed on 10 cm of solid water to allow for sufficient backscatter as observed in standard reference conditions under which the PDD data was taken.

In order to confirm the findings at CSU, similar electron irradiation experiments were carried out at Gifu University (Gifu, Japan). The linear accelerator (Primus Mid-Energy, Siemens Healthcare, Malvern, PA) produced electron beams at 3 MeV and 7 MeV and irradiated cells in identical flasks to the ones used at CSU. In addition to repeating and verifying the CSU results, additional experiments were done with the gantry offset 2 degrees from vertical to investigate the angular dependence of dose distribution due to the flask walls.

In a separate experiment, ultraviolet light exposure was carried out with Phillips germicidal (ultra violet -c) UVC lamps (Phillips, Andover, MA) with dose rate of 1 J/m^2^ per second. Dosimetry was carried out using a UVP UVX dosimeter with UVC probes (UVP, Upland, CA). Cells cultured on P-60 dishes were exposed to UVC after (Phosphate-buffered Saline) PBS wash. Cell culture dishes were rotating with 8 rpm during UVC exposure.

### Cell culture

Chinese hamster ovary (CHO) wild type CHO10B2 was kindly provided by Dr. Joel Bedford at CSU^15^ and maintained at 37 °C in a 5% CO_2_ incubator in Eagle’s minimal essential medium with alpha modification supplemented with 10% heat-inactivated fetal bovine serum, penicillin and streptomycin. Cell doubling times was approximately 12 hours. Cells were maintained in sub confluent exponential growth condition.

**Figure 1:**
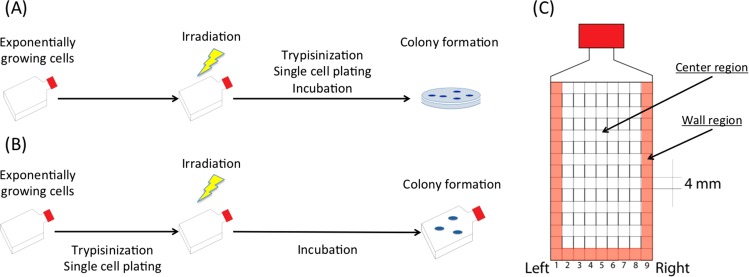
Comparative colony formation assays. In the standard assay (**A**) cells are grown in an incubator, irradiated, trypsinization, and then are re-plated. Whereas in the alternative assay, (**B**), the trypsinization and re-plating is done before irradiation. (**C**) Spatial differences in colony formation was evaluated by dividing the flask into center and wall regions containing 4 × 4 mm^2^ squares where colonies where counted and scored.

For geometric analysis, T25 flasks marked with a measuring grid spacing of 4 mm (Greiner Bio-One, Monroe, NC) were used (Fig. 1). There are 9 squares across left to right. The peripheral region next to wall is categorized as “wall” and the remaining region is categorized as “center” for geometrical analysis.

### Colony formation assay

Two types of cell survival assays were carried out. The first type produced survival curves obtained by a standard colony formation assay as previously described with irradiation followed by single cell plating^16,17^. Briefly, exponentially growing cells in T25 flasks were irradiated, followed by trypsinization, and then the single cell suspension was plated to dishes and incubated for seven days to allow formation of approximately 100 colonies. After the seven days of incubation, the cultures were fixed with 100% ethanol and stained with 0.1% crystal violet. Finally, colonies containing more than 50 cells are scored as survivors.

**Figure 2:**
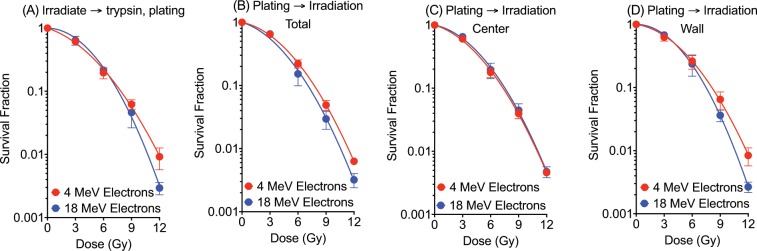
Cell survival curves after 4 and 18 MeV electron irradiation. (**A**) Cell survival curves for cells irradiated using the standard assay and (**B**–**D**) using the alternative assay; (**B**) total counts, (**C**) center region, and (**D**) wall region. Error bars indicate the standard error of the means for three independent experiments.

The alternative colony formation assay used 100, 1,000 or 10,000 cells that were trypsinized and plated to T25 flasks three hours before irradiation allowing them to attach to the flasks. Resulting colonies were counted and scored within specific “wall” and “center” regions to examine spatial effects on cell survival (Fig. 1) due to the dose distribution that varied spatially across the flask. Cell survival curves were plotted with linear quadratic regression using GraphPad Prism 6 software (GraphPad, Ja Lora, CA) (Fig. 2).

**Figure 3:**
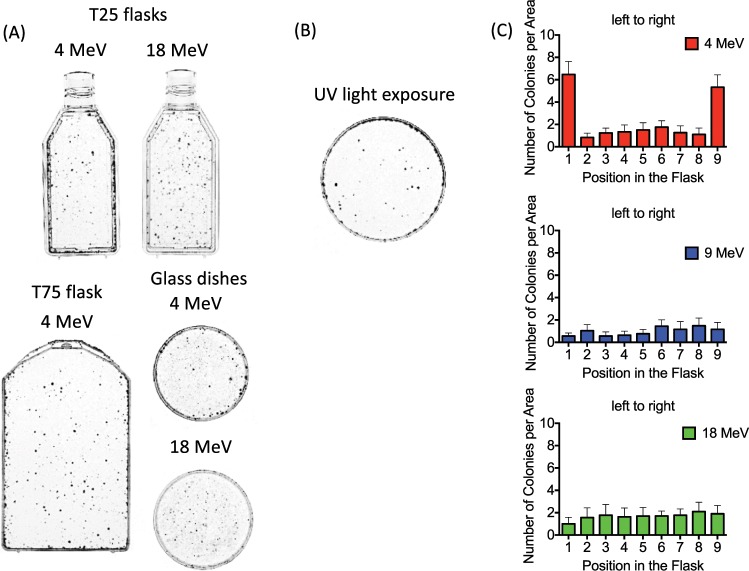
Analysis of colony distributions after 12 Gy at CSU. (**A**) Uneven colony distribution with increased survival near the wall of the flasks for 4 MeV compared to 18 MeV electron irradiation. 100,000 cells were plated to T25 flasks, T75 flask and glass dishes. (**B**) Increased colony survival near the edge of the 6 cm plastic dish after 25 J/m^2^ of UV-C exposure. (**C**) Quantitative analysis of colony distribution using 4, 9, and 18 MeV. 100,000 cells were plated. Error bars indicate the standard error of the means for three independent experiments. Note the increased number of surviving colonies near the edges of the flask at 4 MeV.

To amplify the ability to visualize spatial colony formation differences between beam energies; 100,000 cells were plated using the alternative assay and irradiated with 12 Gy. This was carried out using T25 flask, the T75 flask (Greiner Bio-One), P-60 6 cm dish (Greiner Bio-One), 6 wells plate (Greiner Bio-One), and Pyrex 60 mm glass dish (Pyrex, Corning, NY) in order to remove the flask size and shape influence (Fig. 3A). This was also analyzed using UV light (Fig. 3B). After fixing and staining, locations of colonies were counted within 4 × 4 mm^2^ squares from left to right within the T25 flasks (Fig. 3C) to quantify the spatial survival differences.

A high resolution colony distribution analysis was also performed where the distribution of colonies in the grids on the edge of the flasks were subcategorized into 0–1 mm, 1–2 mm, 2–3 mm, and 3–4 mm from the wall.

### DNA double strand break analysis

DNA double strand break formation was also analyzed by gamma-H2AX foci formation assay with relatively low dose^18^. In brief, confluent CHO cells were irradiated; 30 min after irradiation, cells were washed with PBS and fixed with 4% paraformaldehyde in PBS for 15 minutes, followed by treatment with 0.2% Triton X-100 solution in PBS for 5 minutes. Before immunocytochemical detection of γ-H2AX, cells were blocked with 10% goat serum solution for one hour at room temperature or overnight at 4 °C. Mouse monoclonal antibody for anti-γ-H2AX (Millipore, Billerica, MA) was diluted 1:500 and treated for one hour at 37 °C. Cells were then washed with PBS three times for 10 minutes each, and the secondary antibody (Alexa Fluor 488 conjugated goat anti-mouse, Invitrogen) diluted 1:500 was added, and the slides were incubated for one hour at 37 °C. Flasks were sawed and mounted in a solution of 1.5 μg/ml DAPI containing slow-fade (Invitrogen) after four washes with PBS for 10 minutes each. Fluorescence images were captured using a Zeiss Axioskop fluorescent microscope (Zeiss, Jena, Germany) equipped with Q-imaging Aqua Camera (Q-imaging, QC, Canada) with Q-capture pro software. Three independent experiments were performed. Manual counting was performed for 50 cells to obtain average foci number per cell.

### Radiochromic film analysis

The spatial dose distribution was measured at CSU using EBT3 Gafchromic film. Three sets of measurements were carried out with film: (1) open field, (2) film placed directly underneath the flasks with lids, and (3) without lids. In all sets the same nominal energies were used (4, 9, and 18 MeV), and under the same conditions as was done when the cells were present with the same volume of media within the flasks. For each energy, the number of monitor units (MU) was calibrated to account for energy specific dose build up; 115, 121, 106 for 4, 9, 18 MeV respectively. This was the corrected MU value to deliver 100 cGy to the surface of the flasks, under 2 mm of media, where the cells were attached.

### Monte carlo analysis

As a confirmation of origin to the nature of the altered dose distributions, electrons were tracked within the flask wall using Monte Carlo simulations (EGSnrc Tutor7pp from The National Research Council of Canada)^19^. Tutor7pp is a standard application provided with the EGSnrc package of electron transport in matter codes. The geometry of the flask object in the simulation was matched to the geometric proportions and materials of a single wall of a flask having a two-millimeter layer of media on the floor divided into two segments for dose scoring. In addition, a large water phantom was created within the model to account for backscatter in accordance to the cell experiment and film measurements. Transport parameters were modified to allow for photoelectric angular scatter, electron impact ionizations, atomic relaxations, and Rayleigh scattering, with an electron energy cutoff of 1 keV. Scatter from the flask walls was modeled using Monte Carlo simulations from a point-source beam of 1000 monoenergetic electrons of energies: 4 MeV, 9 MeV, and 18 MeV impingent directly upon the wall of the flask from directly above. Electrons were allowed to scatter into the flask wall, media, water phantom, and interior air cavity, but not outside the flask to preserve clarity. Figures were generated using the C++ geometry viewer egs_view.

### Statistical analysis

Statistical analysis was carried out with GraphPad Prism 6 for student T-test (two-ways) or one-way ANOVA. All experiments were conducted independently at least three times. P values less than 0.05 were considered to be statistically significant.

## Results

### The distribution of colonies

The standard colony formation assay (irradiation followed by trypsinization, then plating single cells to form colonies) was carried out to determine cellular radiosensitivity to different energies of electrons. The cell survival curves showed at high doses decreased cellular radiosensitivity at 4 MeV electrons compared to cells irradiated at 18 MeV electrons (Fig. 2A). The survival fractions after 12 Gy of electron beam were 0.01 for 4 MeV and 0.003 for 18 MeV. The difference was statistically significant (t-test, p < 0.05).

The alternative colony formation assay also resulted in differences between cells irradiated with 4 MeV and 18 MeV after 12 Gy of irradiation, but survival was not statistically significant over the entire region of the flask (Fig. 2B). However, the surviving colonies exposed to 4 MeV tended to have a higher concentration on the peripheral edges (Fig. 3A). When spatial dependence was considered by comparing the flask walls and centers, and found cell survival fractions were significantly different (p < 0.05) at wall for 4 MeV and 18 MeV electrons (Fig. 2D). Conversely, cells in the center demonstrated statistically the same degree of radiation sensitivity (Fig. 2C).

Uneven colony distribution could be visually observed, with increased survivors near the edge of the flask when 4 MeV electrons were used compared to 18 MeV. This was observed not only for the T25 flasks, but also T75 flasks, and P-60 mm glass dishes (Fig. 3A), as a positive control CHO cells exposed to 25 J/m^2^ of UVC and showed enhanced survival on the peripheral area (Fig. 3B). Further investigation of spatial dependence of survival within the flasks used 4 × 4 mm grids for 4, 9, and 18 MeV of electrons. Flasks irradiated with 4 MeV showed significant (ANOVA, p < 0.05) survival at the wall compared to the center (Fig. 3C).

**Figure 4:**
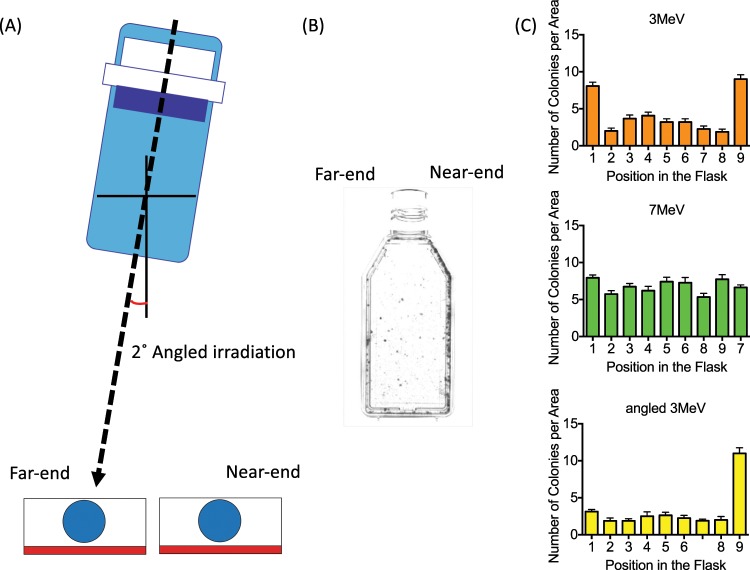
Analysis of colony distributions after 12 Gy of 3 and 7 MeV electron irradiation at Gifu University. (**A**) The gantry was angled 2° during irradiation. (**B**) Visualization of uneven colony distribution after angled irradiation. (**C**) Quantitative analysis of uneven colony distribution after 3 and 7 MeV electron irradiation and 3 MeV electron irradiation with 2° gantry angle. Error bars indicate the standard error of the means for three independent experiments. Note the similar outcome as at CSU where colonies near the edges had an increased surviving number of colonies. The angled gantry also spared colonies that were shielded by the flask wall.

In order to confirm the findings of the uneven distribution of colonies, similar experiments were also carried out at Gifu University using 3 MeV and 7 MeV electron energy beams on an identical flask apparatus. Indeed, 3 MeV electron beams yielded uneven colony distribution in the flask, but not for the higher energy 7 MeV electron beam (Fig. 4) supporting our earlier findings. Additionally, irradiation with 3 MeV electrons was carried out with the gantry offset 2° from vertical to investigate the angular dependence of the incident beam and the flask walls (Fig. 4B). With angled irradiation, uneven colony formation was observed only on the ‘near-end’ side of the flask (Fig. 4C) where scatter from the flask wall was reduced.

### DNA damage distribution analysis

**Figure 5:**
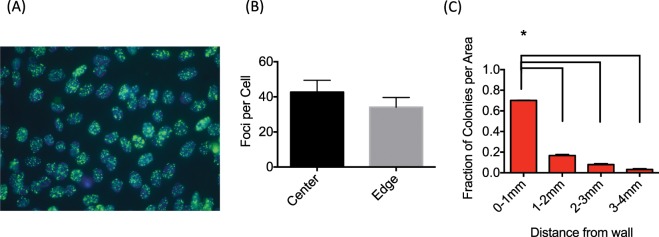
The amount of DNA damage after 4 MeV electron irradiation. (**A**) an example of gamma-H2AX foci after 1 Gy of 4 MeV electron irradiation for the center area. (**B**) Quantitative analysis of uneven foci distribution that was not significantly different between the center and edge areas. (**C**) Colony distribution after 4 MeV electron irradiation at the edge of the flask. Error bars indicate the standard error of the means for three independent experiments. * indicates statistically significant differences (ANOVA, p < 0.01).

Uneven colony formation distribution due to heterogeneous dose distribution were also quantified by measuring DNA double strand breaks using gamma-H2AX foci formation assay. After 1 Gy of 4 MeV electron irradiation, cells located on the center region (Fig. 1B, flask area between 2 and 8) had 42.7 foci per cells and cells located within 4 mm from edge (Fig. 1B, flask area 1 and 9) had 34.1 foci per cells (Fig. 5). The difference was not statistically significant (p = 0.199) but on average approximately 20% reduction of foci was observed at the edge of flasks compared to the center area.

The colony distribution was re-examined through a high resolution spatial sampling to determine where the uneven DNA damage distribution occurred. We used 1 mm increment distances from flask wall, up to 4 mm (one grid). These measurements showed that 50% of colonies were formed within 0–1 mm from edge of the flask (Fig. 5C).

### Dose distribution gafchromic film analysis

**Figure 6:**
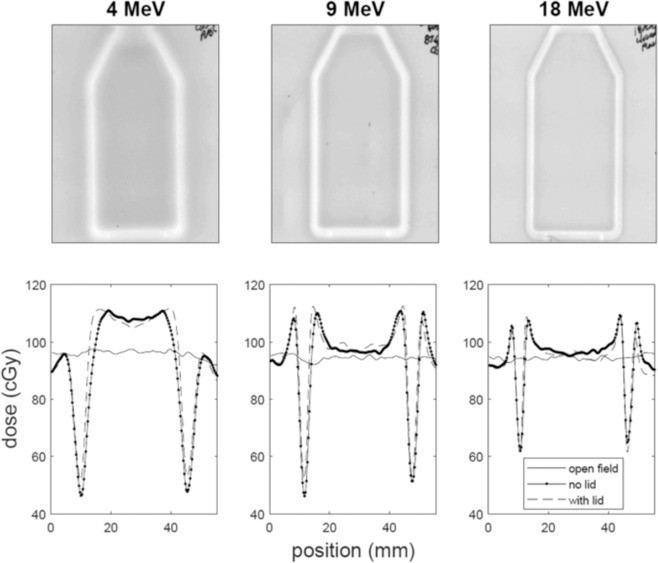
Film measurements taken with EBT3 Gafchromic film. The upper row shows the raw exposed film images for the three electron energies. The lower row gives the calibrated dose for open field (solid line), with the flask in place without the lid (point line), and with the flask in place with the lid (dashed line) as was done during the cell irradiation experiments.

The physical dose distribution was measured with Gafchromic film located either in open field, or under of the flasks with or without the flask lip in place. Qualitatively, the scatter from the flask walls was more pronounced at low energies, and tends to reduce dose near the wall while increasing the dose within the center of the flask due to the additive scattered dose to the primary beam. This effect is drastically reduced when increasing the electron energy to 9 MeV and further reduced in the 18 MeV case. The dose reduction as a function of distance from wall can also be seen to be less sensitive to scatter as the energy of the electron beam is increased. The dose near walls of the flask showed a maximum decrease of approximately 65% dose compared to the center of flasks in 4 MeV electron study compared to 40% in the 18 MeV electrons (Fig. 6).

### Monte carlo scatter predictions

**Figure 7:**
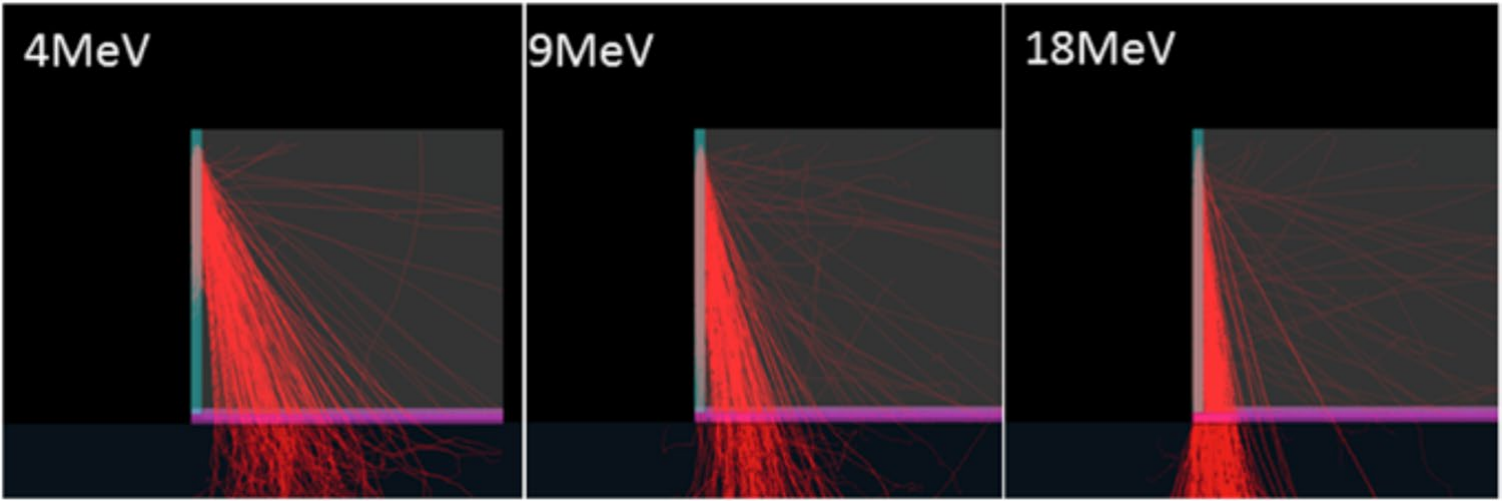
Monte Carlo simulation to visualize primary electron tracks (red) scattered from flask wall for the same energies used at CSU. Note the increased lateral electron scatter from the plastic (teal) wall at lower energies. Also included in the simulation was PBS media (magenta) to align 2 mm of the bottom of the flask, air (gray), and solid water (grey) placed under the flask as was done in the experimental setup.

Monte Carlo scatter predictions also show an energy dependence of the electron scattering and interaction with the wall material (Fig. 7). Low energy electrons have wider scattering distribution than higher energy and are visibly scattered away before reaching the surface of the flask creating a large zone of reduced dose directly adjacent to the flask wall. Additionally, attenuation of the electron range is greater in low energy electrons which can be seen in the color differential of the wall material. The combination of the larger scattering angle and the attenuation in the low energy beam decreases the population of electrons that scatter close to the wall. The model indicates that there is a substantial gap at the base of the wall in the 4 MeV case. As the energy of the electrons increase, the scatter increases in the forward direction, leading to an increase in dose where the primary and scattered electrons combine. This effect was most prominent in the 18 MeV model. The 9 MeV case shows an intermediate result between these two extremes. This simulation agrees with the dose deposition film measurement and visually demonstrates the energy dependent scattering behavior.

## Discussion

Dose uniformity is important in conducting accurate and reproducible experiments between institutions. Dose distribution is a well-researched problem in ultraviolet light exposure (UV shadowing)^20^. Uneven dose distribution often occurs in UV irradiation because the wavelength is easily absorbed by plastic cell culture vessels. A rotating sample tray irradiated by vertical UV source may alleviate uneven distribution. Nevertheless, vessel walls can easily shield cells from UV exposure and inhomogeneous dose distributions can lead to uneven colony distributions after UV exposure (Fig. 3B). High energy radiation is also affected by scatter within vessel walls, and has previously been demonstrated in 6 and 15 MV X-rays^21^ and found dose uniformity hard to predict when irradiating cell flasks, noting the importance of spatial dosimetry measurements to be done in parallel.

We have found the scattering of MeV electrons can also be greatly affected by the flask walls, particularly at low energies due to increased lateral scatter and an interaction with flasks. This can often lead to misleading interpretations in cell survival irradiation experiments. Importantly, because irradiation procedures from varied labs are unclear, unspecified, and untraceable regarding irradiation protocols and verification of the spatial dose distribution, results and conclusions drawn from varying scientific papers should be regarded carefully^22–27^.

The dose uniformity presented in this study was mostly altered at the lowest electron energies (3 and 4 MeV, Figs. 3 and 4). Increased colony formation was often observed just next to the walls within the 4 mm measuring grids due to the lower dose along the flask wall. The gamma-H2AX foci experiments did not show a statistically significant difference between the center and wall regions. This was likely due to the analysis region being relatively large (4 × 4 mm) in area, leading to an averaging of damage between the low dose proximal wall region and the higher dose remaining area which was confirmed with smaller scale colony analysis (Fig. 5C). It is also possible that the higher doses were required to get statistical significance for gamma-H2AX assay as cell survival experiments used 12 Gy to observe a large difference. However, gamma-H2AX foci counting has a limitation for higher doses. The gamma-H2AX DNA damage assay indicated approximately a 20% reduction within the 4 mm proximal grid unit compared to the center region. Gafchromic film analysis confirmed a reduced dose near the wall edge as well as increased dose near the center due to the combined scatter beam from the wall with the primary beam. Monte Carlo simulations also confirmed the increased lateral scattering profile with decreasing energy (Fig. 7).

The obliquity of the electron beam was also critical. When the electron beam central axis was angled by 2°, the distal side had fewer survivors that could be seen from visual inspection (Fig. 4). This suggests that the plastic distal sidewall of flask scattered electrons back onto cells compared to the proximal side that was partially shielded and received lower dose.

We believe findings within this study suggest that conclusions should be drawn more carefully regarding the relative radioresistance after high dose measurements of cell survival using low energy electrons. When evaluating the entire flask without considering the spatial dose distribution, there was a statistically significant increase in cell survival for 4 MeV suggesting less radiosensitivity at low electron energies. However, after incorporating the spatial dose corrections and considering only the center of the flask where uniform dose was measured, the cell survival counts for 4 MeV and 18 MeV electron EBT were essentially identical (Fig. 2C). This result agreed the previous researches for the similar relative biological effectiveness among 2–20 MeV electrons^5^. Therefore, previous interpretations of radiosensitivity to the low energy electrons may need to be reanalyzed to carefully consider uneven dose distribution due to the scattering from the flask walls.

In order to minimize uneven dose distribution effects and to estimate the true radiosensitivity to low energy electrons, cells should be irradiated after trypsinization, counting, and plating and only colonies on the center area within a uniform radiation field should be scored as survivors. Ideally, confirmation of the dose distribution within publications would be included. Alternatively, after irradiation, cells near the edge of vessels should be mechanically removed by wiping or scraping before trypsinization for single cell plating.

In conclusion, our data demonstrates that increased lateral scattering found at low energy electrons can strongly affect the dose delivered near the cell walls due to cell wall interactions to the cells in the culture vessels. The reduced dose can result in underestimating cellular radiosensitivity, especially at low electron therapeutic energies. The uniformity of dose distribution should be carefully considered for research involving cell survival studies within flasks with electron beams.
